# A Retrospective Comparison of Transcutaneous and End-Tidal Carbon Dioxide Monitoring in Pediatric Polysomnography

**DOI:** 10.7759/cureus.109308

**Published:** 2026-05-20

**Authors:** Tak Kong Tsui

**Affiliations:** 1 Pediatrics, Pamela Youde Nethersole Eastern Hospital, Hong Kong, CHN

**Keywords:** end-tidal co2 monitoring, nocturnal hypoventilation, obstructive sleep apnoea, paedatrics, polysomnography, trancutaneous co2 monitoring

## Abstract

Background

Accurate monitoring of carbon dioxide (CO₂) is important in pediatric polysomnography (PSG), particularly for identifying nocturnal hypoventilation, a condition with significant clinical consequences in children, especially those with neuromuscular or pulmonary disorders. Two primary modalities are available for non-invasive CO₂ estimation: end-tidal CO₂ (EtCO₂) and transcutaneous CO₂ (TcCO₂). While both are widely used, their diagnostic equivalence remains unclear. This study aimed to address the clinical question of whether TcCO₂ and EtCO₂ measurements in pediatric PSG can replace each other.

Methods

A single-center retrospective study was conducted by including pediatric patients who underwent PSG with concurrent EtCO₂ and TcCO₂ monitoring. For each patient, mean, maximum, and minimum CO₂ values, as well as sleep duration with values exceeding 50 mmHg, were extracted and analyzed between the two modalities. The diagnostic rates of nocturnal hypoventilation as defined by the American Academy of Sleep Medicine (AASM) Manual were also compared.

Results

Among 127 children (median age 11 years, interquartile range age eight to 14), based on the Bland-Altman analysis, a positive mean bias was observed in all three comparisons, suggesting that EtCO₂ measurements were systematically lower than TcCO₂. Wide 95% limits of agreement in all TcCO₂ and EtCO₂ measurements were observed. A weakly positive correlation but with marginal statistical significance was noted between mean TcCO₂ and EtCO₂ values (rs=0.173, p=0.052). Maximum TcCO₂ and EtCO₂ values were also only weakly correlated (rs=0.288, p=0.001). The median of mean TcCO₂ was significantly higher than that of mean EtCO₂: 42.95 mmHg vs. 39.00 mmHg, respectively (Z = −8.06, p < 0.001). The median of maximum TcCO₂ was also significantly higher than that of maximum EtCO₂ values: 47.40 mmHg vs. 44.00 mmHg, respectively (Z = −7.50, p < 0.001). However, no statistically significant difference was observed for the corresponding minimum values (36.60 mmHg vs. 35.00 mmHg; Z = −1.94, p = 0.052). Ten out of 127 (7.9%) patients were diagnosed with hypoventilation by TcCO₂ measurement, none of whom could be diagnosed based on EtCO₂ measurement. Subgroup analysis by age, sex, obstructive sleep apnea (OSA) severity, obesity, craniofacial abnormalities, tonsillar hypertrophy, and syndromic diagnosis yielded no significantly different findings.

Conclusions

Our study suggested that EtCO₂ and TcCO₂ measurements in pediatric PSG may not be interchangeable, given the observed suboptimal agreement between the two modalities, i.e., the tendency for TcCO₂ values to be higher than EtCO₂, and the weak and mostly statistically insignificant correlation observed for CO₂ measurements. However, in the absence of arterial blood gas (ABG) as a reference standard, it was not possible to determine which modality more accurately reflects true arterial CO₂ levels or was more reliable for identifying nocturnal hypoventilation.

## Introduction

Sleep-related breathing disorders are important conditions in the pediatric population, ranging from primary snoring to obstructive sleep apnea (OSA) and nocturnal hypoventilation [1]. Accurate assessment of ventilation during sleep is therefore an essential component of pediatric polysomnography (PSG). In addition to monitoring airflow and oxygen saturation, measurement of carbon dioxide (CO₂) levels provides invaluable information regarding the adequacy of ventilation during sleep. The American Academy of Sleep Medicine (AASM) defines hypoventilation as arterial partial pressure of carbon dioxide (PaCO₂) or its surrogate exceeding 50 mmHg for at least 25% of the total sleep time in the pediatric population [2]. Apart from OSA, children with conditions such as neuromuscular disorders [3-5], congenital central hypoventilation syndrome (CCHS), obesity hypoventilation syndrome (OHS) [6], and underlying pulmonary disease are particularly at higher risk of nocturnal hypoventilation. Current scoring guidelines from the AASM emphasize the importance of including CO₂ monitoring in the evaluation of sleep-related hypoventilation during pediatric PSG [2].

Currently, there are two commonly used methods available as surrogates for arterial PaCO₂ monitoring during PSG [2]. End tidal carbon dioxide (EtCO₂) monitoring measures the PaCO₂ in exhaled gas at the end of expiration and is commonly used as a non-invasive estimate of alveolar CO₂ tension. One advantage of EtCO₂ monitoring is that it provides breath-by-breath measurements and therefore allows rapid detection of changes in ventilation. However, the accuracy of EtCO₂ measurements can be influenced by physiological and technical factors, such as mouth breathing, physiological dead space, or displacement of nasal sampling cannulae [7].

Transcutaneous carbon dioxide (TcCO₂) monitoring provides an alternative non-invasive approach for assessing CO₂ levels. This technique estimates CO₂ tension by measuring the diffusion of CO₂ across the skin using a heated electrode, which increases local blood flow and facilitates diffusion of carbon dioxide from arterialized capillary blood [8]. As a result, TcCO₂ measurements tend to approximate arterial CO₂ tension more closely and can provide a continuous estimate of systemic CO₂ levels [8]. TcCO₂ monitoring has therefore become increasingly utilized in pediatric sleep laboratories for the detection of nocturnal hypoventilation and is recommended as a useful adjunct in pediatric PSG. Nevertheless, TcCO₂ measurements may also be influenced by factors such as skin perfusion, sensor temperature, and the inherent delay associated with diffusion-based measurements [8].

Given the different physiological principles underlying these two monitoring techniques, discrepancies between TcCO₂ and EtCO₂ measurements may occur in PSG. Previous studies in pediatric and adult populations have demonstrated that TcCO₂ values often exceed EtCO₂ values in studies related to CO₂ monitoring with PSG [9-10]. Despite the increasing use of both monitoring modalities in pediatric PSG, the clinical interchangeability of TcCO₂ and EtCO₂ measurements remains an area of ongoing investigation. 

In this study, we aimed to address the clinical question of whether TcCO₂ and EtCO₂ measurements in pediatric PSG can replace each other. Specifically, this study has the following objectives. The primary objective was to assess the interchangeability of TcCO₂ and EtCO₂ measurements during pediatric PSG. The secondary objectives were to assess the correlation and systematic bias between the two modalities and their respective diagnostic utility in detecting nocturnal hypoventilation.

## Materials and methods

Study design and settings

This study was a retrospective cross-sectional analysis conducted at the Pamela Youde Nethersole Eastern Hospital, a tertiary pediatric sleep center in Hong Kong, China, evaluating PSG studies performed between 1st January 2023 and 31st December 2024. Ethics approval was obtained from the Hospital Authority Central Institutional Review Board (approval no. PAED-2026-024.4), and all data were de-identified prior to analysis.

At the study center, full PSG was performed using the Philips Respironics Alice 6 system (Philips Respironics, Murrysville, PA, USA), and all component setups followed the recommendations in the AASM Manual for the Scoring of Sleep and Associated Events [2]. Simultaneous TcCO₂ and EtCO₂ were monitored in each patient. The TcCO₂ monitoring was performed using an electronic sensor placed on well-perfused areas of the patients’ infraclavicular region using the Sentec Digital Transcutaneous CO₂ monitoring system (Sentec, Therwil, CHE). The TcCO₂ monitoring sensors were changed every four hours to prevent sensor drift and thermal injury. Two-point calibration was obtained following the protocol before and after each sleep test. The corresponding readings were recorded and analyzed by the Sentec V-STATS software. The TcCO₂ data were processed using Sentec V-STATS software, which applied a linear drift correction based on pre- and post-measurement calibrations to ensure data stability and accuracy, and drift-corrected data were used in the data analysis. The EtCO₂ measurements were obtained via a nasal pressure transducer connected to the Alice 6 system. Precise temporal alignment of TcCO₂ and EtCO₂ signals was technically restricted by the inherent physiological lag of transcutaneous diffusion, which created a variable phase shift that precluded reliable epoch-by-epoch synchronization.

All PSG records in this study were scored by two highly experienced Registered Polysomnographic Technologists (RPSGT) according to the AASM manual. To maintain quality control, a specialist in pediatric respiratory medicine audited all of the studies to ensure scoring consistency. Internal audit showed a high level of agreement (>90%) with the primary scoring. Patients who failed CO₂ monitoring due to technical reasons, such as intolerance of the TcCO₂ probe or nasal pressure transducer, significant mouth breathing, or signal artifacts, were identified. Any periods of technical artifact (e.g., sensor displacement, cannula blockage, or moisture in the sampling line) were identified through visual inspection of the waveforms and device-specific quality indicators. Periods where either the TcCO₂ or EtCO₂ signal was absent or deemed poor quality by the scoring technologist were excluded from the final analysis.

The inclusion criteria of this study were pediatric patients aged three to 18 years who underwent diagnostic PSG studies with concurrent EtCO₂ and TcCO₂ monitoring. Patients who underwent PSG for positive airway pressure (PAP) titration, those who had significant missing data in TcCO₂ or EtCO₂ monitoring, or failed TcCO₂ calibration or probe intolerance, were excluded from the study.

Patient data extraction

The patients were identified from the records in the computerized clinical record system. Their baseline demographics were retrieved, which included age, sex, presence of obesity defined as BMI equal to or more than the 95th percentile for age and sex, presence of tonsillar hypertrophy, presence of craniofacial abnormalities, and any syndromic diagnoses. The PSG parameters of patients were retrieved from the computer system, including the obstructive apnea-hypopnea index (OAHI) per hour of total sleep time (TST), the nadir of oxygen saturation (SpO₂) by pulse oximetry, and the arousal index. Grading of OSA severity was primarily based on the values of OAHI. For patients aged below 13, OSA severity grading was defined as the following: mild (OAHI 1-4/hr TST), moderate (OAHI 5-10/hr TST), or severe (>10/hr TST) [11]. For patients aged 13 or above with an adult body habitus, the adult criteria for grading of OSA severity were adopted instead: mild (OAHI 5-15/hr TST), moderate (OAHI 15-30/hr TST), and severe (OAHI >30/hr TST) [12].

Results for CO₂ measurements from both monitoring modalities (TcCO₂ and EtCO₂) were retrieved, including mean, maximum, and minimum values and percentage of time with TcCO₂ and EtCO₂ exceeding 50 mmHg. Hypoventilation was defined based on the AASM manual: surrogate of arterial PaCO₂ (TcCO₂ or EtCO₂) exceeding 50 mmHg for at least 25% of TST [2]. The diagnostic rate of nocturnal hypoventilation using both monitoring modalities was then compared. 

Statistical analysis

All statistical analyses were performed using IBM SPSS Statistics version 29.0.0.0 (IBM Corp., Armonk, NY, USA). The agreement between TcCO₂ vs. EtCO₂ values was analyzed using the Bland-Altman analysis [13]. The mean bias and limits of agreement (LoA) were calculated. Based on prior clinical studies on the comparison of various CO₂ measurement modalities, including TcCO₂, EtCO₂, and PaCO₂, in the pediatric population, we adopted the definition of clinical interchangeability as a mean bias within ±5 mmHg and LoA with ±5 mmHg [14-16]. Spearman’s rank correlation was used to test for correlation between mean, maximum, and minimum TcCO₂ and EtCO₂ values. The Wilcoxon signed-rank test [17] was used to compare for systematic differences between mean, maximum, and minimum TcCO₂ and EtCO₂ values. Statistical significance was defined as a p-value < 0.05.

## Results

Study population

A total of 138 pediatric PSG studies were identified during the study period. Eleven cases (8%) were found to have failed EtCO2 monitoring due to technical issues and were excluded, whereas no patients had failed TcCO2 monitoring. Only 127 patients were included in the final analysis. There were no missing values for baseline variables.

Baseline demographic characteristics

Baseline demographic characteristics of the study population are listed in Table 1. The study population was predominantly male, comprising 91 males (71.7%) and 36 females (28.3%). Participant age ranged from three to 18 years, with a median age of 11 years (Interquartile range (IQR) eight to 14 years), mean age (+/- standard deviation (SD)) 11 years ± 3.9. Sixty-two patients (48.8%) met criteria for obesity. Tonsillar hypertrophy was present in 68 patients (53.5%). Craniofacial abnormalities were identified in only 21 patients (16.5%). Syndromic diagnoses were uncommon in our study, involving only three patients (2.4%), with one case of Prader-Willi syndrome, Dravet syndrome, and fibrodysplasia ossificans progressiva, respectively. Overall, our patients were predominantly male, school-aged/adolescent, and around half had tonsillar hypertrophy and obesity, but a smaller proportion had craniofacial abnormalities or syndromic conditions.

**Table 1:** Baseline demographic characteristics of the study population (n = 127) Data are presented as mean +/- SD and median (IQR) for continuous variables, and number (percent) for categorical variables. All patients had complete baseline data. IQR: Interquartile range

Characteristic		Number
Age (in years)	Median (IQR)	11 (8-14)
	Mean ± SD	11.0 ± 3.9
Sex	Male	91 (71.7%)
	Female	36 (28.3%)
Obesity	Present	62 (48.8%)
	Absent	65 (51.2%)
Tonsillar hypertrophy	Present	68 (53.5%)
	Absent	59 (46.5%)
Craniofacial abnormality	Present	21 (16.5%)
	Absent	106 (83.5%)
Syndromic diagnosis	Present	3 (2.4%)
	Absent	124 (97.6%)

Baseline PSG characteristics

The study population exhibited a wide range of severity of sleep-disordered breathing (Table 2). The OAHI showed a right-skewed distribution, with a mean of 10.1 ± 13.8 events/hour, a median of 4.4 events/hour, and an IQR of 1.7-13.1. The OAHI values ranged from 0 to 75.8 events/hour, indicating substantial heterogeneity in disease severity.

**Table 2:** Polysomnographic characteristics of the study population (n = 127) OAHI: Obstructive apnea-hypopnea index, SpO₂: Oxygen saturation

Characteristic	Range	Mean ± SD	Median (IQR)
OAHI, events/hour	0 to 75.8	10.1 ± 13.8	4.4 (1.7-13.1)
SpO₂ nadir, %	54 to 99	88.0 ± 6.8	89.0 (85.0-93.0)
Arousal index, events/hour	1.7 to 51	8.6 ± 7.3	6.5 (5.0-10.2)

The mean nocturnal SpO₂ nadir was 88.0 ± 6.8%, with a median of 89.0% and an IQR of 85% to 93%. The distribution was negatively skewed, with values ranging from 54% to 99%. The arousal index also demonstrated a right-skewed distribution, with a mean of 8.6 ± 7.3 events/hour and a median of 6.5 events/hour (IQR 5.0-10.2), ranging from 1.7 to 51.0 events/hour.

Categorization of the OSA severity per the OAHI is tabulated in Table 3. Thirty-eight patients (29.9%) did not meet the criteria of OSA, 47 (37%) were of mild grade, 16 (12.6%) of moderate grade, and 26 (20.5%) of severe grade.

**Table 3:** OSA severity distribution by OAHI (n=127) OSA: Obstructive sleep apnea, OAHI: Obstructive apnea-hypopnea index

OSA severity	Number (percentage)
No OSA	38 (29.9%)
Mild	47 (37.0%)
Moderate	16 (12.6%)
Severe	26 (20.5%)

Agreement between TcCO₂ and EtCO₂ measurements

Mean TcCO₂ and EtCO₂ Measurements

**Figure 1:**
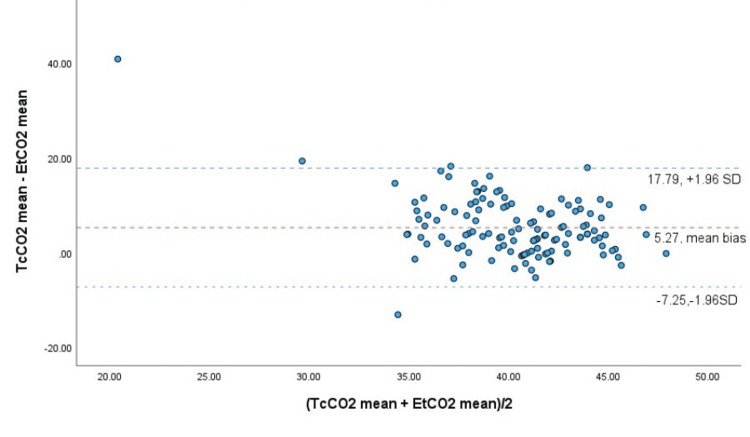
Bland-Altman plot showing agreement between mean TcCO₂ and mean EtCO₂ measurements The red dashed line represents the mean bias (+5.27 mmHg), and the blue dashed lines indicate the upper and lower 95% limits of agreement (−7.25 to +17.79 mmHg). TcCO₂: Transcutaneous CO₂, EtCO₂: End-tidal CO₂, CO₂: Carbon dioxide

Bland-Altman analysis demonstrated a systematic bias between mean TcCO₂ and EtCO₂ measurements (Figure 1). The mean bias (TcCO₂ − EtCO₂) was +5.27 (95% CI 4.15-6.39) mmHg, indicating higher TcCO₂ values relative to EtCO₂. The 95% limits of agreement ranged from −7.25 to +17.79 mmHg, exceeding the predefined acceptable range of ±5 mmHg, suggesting poor agreement between the two measuring modalities.

Maximum TcCO₂ and EtCO₂ Measurements

**Figure 2:**
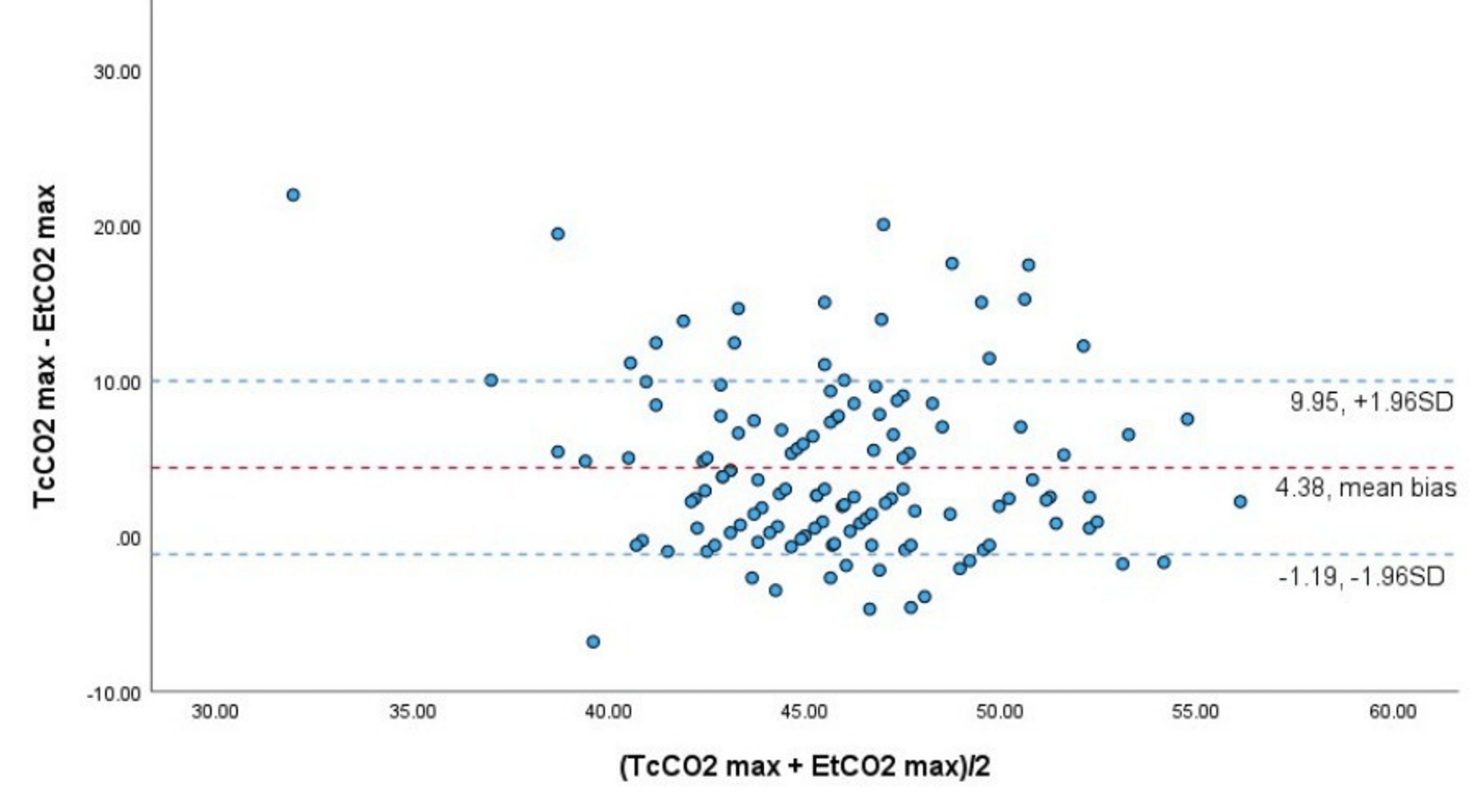
Bland-Altman plot showing agreement between maximum TcCO₂ and EtCO₂ measurements The red dashed line represents the mean bias (+4.38 mmHg), and the blue dashed lines indicate the upper and lower 95% limits of agreement (−1.19 to +9.95 mmHg). TcCO₂: Transcutaneous CO₂, EtCO₂: End-tidal CO₂, CO₂: Carbon dioxide

Similarly, a systematic bias was noted between maximum TcCO₂ and EtCO₂ measurements (Figure 2). The mean bias (TcCO₂ − EtCO₂) was +4.38 (95% CI 3.4-5.36) mmHg, indicating higher TcCO₂ values relative to EtCO₂. The 95% limits of agreement ranged from −1.19 to +9.95 mmHg. While the lower limit fell within the predefined acceptable range of ±5 mmHg, the upper limit exceeded this threshold, suggesting poor agreement between the two measuring modalities.

Minimum TcCO₂ and EtCO₂ Measurements

**Figure 3:**
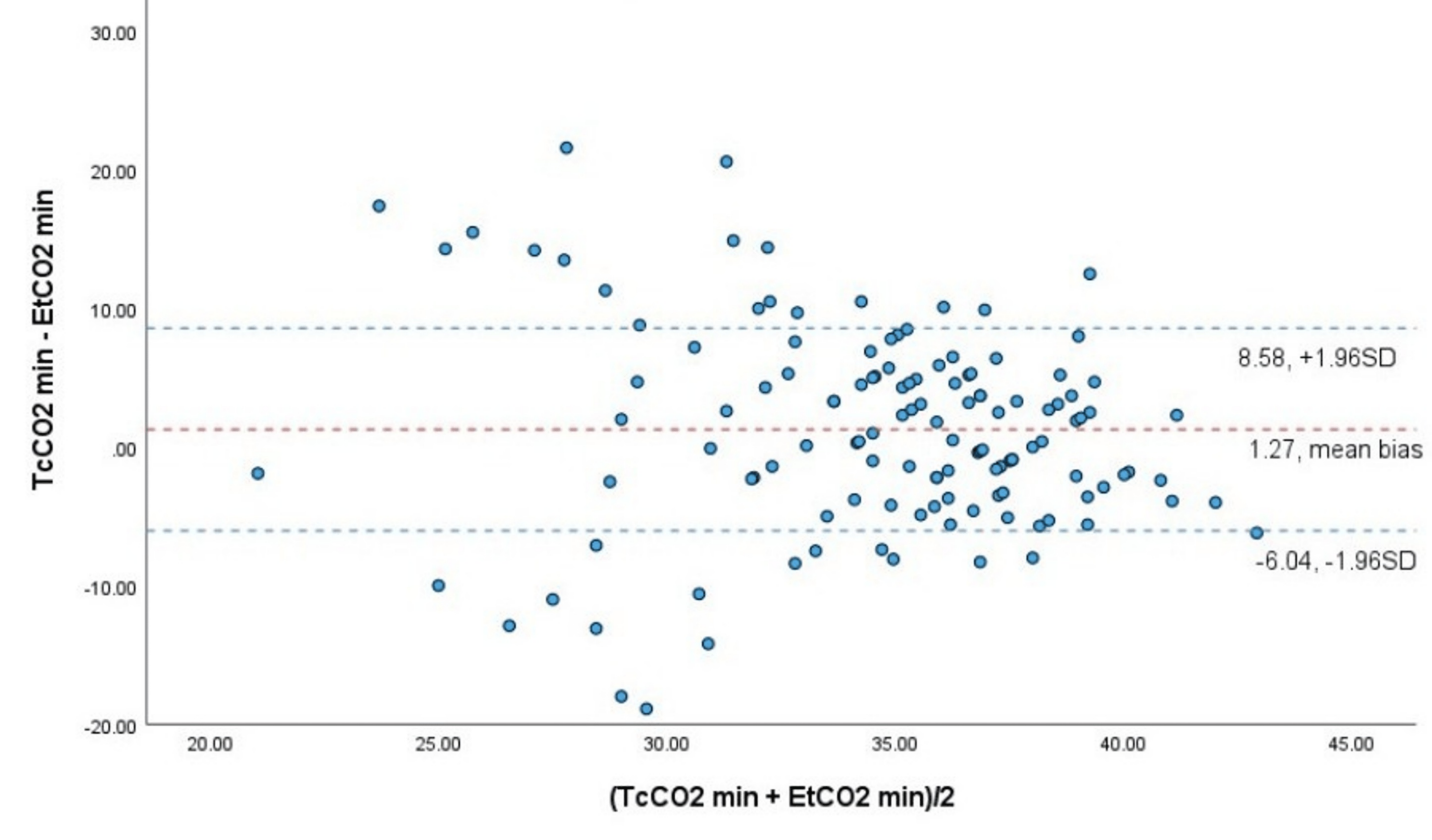
Bland-Altman plot showing agreement between minimum TcCO₂ and EtCO₂ measurements The red dashed line represents the mean bias (+1.27 mmHg), and the blue dashed lines indicate the upper and lower 95% limits of agreement (−6.04 to +8.58 mmHg). TcCO₂: Transcutaneous CO₂, EtCO₂: End-tidal CO₂, CO₂: Carbon dioxide

Finally, the mean bias between minimum TcCO₂ and EtCO₂ measurements (TcCO₂ − EtCO₂) was +1.27 (95% CI −0.01-2.56) mmHg (Figure 3), indicating a lesser extent when compared with mean and maximum TcCO₂ and EtCO₂ measurements. However, the 95% limits of agreement, ranging from −6.04 to +8.58 mmHg, still suggested poor agreement between the two measuring modalities.

The degree of correlation between TcCO₂ and EtCO₂ is listed in Table 4. A weakly positive correlation was noted for mean values; however, the result was of marginal statistical significance (rs=0.173, p=0.052). For maximum TcCO₂ and EtCO₂ values, the correlation was also weak but statistically significant (rs=0.288, p=0.001). Finally, the minimum values of TcCO₂ and EtCO₂ were weakly correlated and not statistically significant.

**Table 4:** Spearman’s rank correlation between TcCO₂ and EtCO₂ values TcCO₂: Transcutaneous CO₂, EtCO₂: End-tidal CO₂, CO₂: Carbon dioxide

Correlation	Spearman coefficient (rs)	p-value
Mean TcCO₂ vs. EtCO₂	0.173	0.052
Maximum TcCO₂ vs. EtCO₂	0.288	0.001
Minimum TcCO₂ vs. EtCO₂	0.11	0.22

The Wilcoxon signed-rank test was used for comparing TcCO₂ and EtCO₂ values due to the non-normal distribution of the data (Table 5). The median of mean TcCO₂ was 42.95 mmHg (IQR 40.50-45.43) compared with 39.00 mmHg (IQR 34.00-41.00) for EtCO₂, demonstrating a statistically significant difference (Z = −8.06, p < 0.001). Similarly, maximum TcCO₂ values were significantly higher than maximum EtCO₂ values (47.40 (IQR 45.00-50.83) vs. 44.00 (IQR 41.00-46.00) mmHg; Z = −7.50, p < 0.001). No statistically significant difference was observed for corresponding minimum values (36.60 (IQR 33.65-38.63) vs. 35.00 (IQR 32.00-38.00) mmHg; Z = −1.94, p = 0.052). Overall, these findings indicated that EtCO₂ measurement yielded lower values for CO₂ levels when compared with TcCO₂ measurement, particularly for mean and peak values.

**Table 5:** Comparison of TcCO₂ and EtCO₂ values using the Wilcoxon signed‑rank test The values are presented as median (IQR). TcCO₂: Transcutaneous CO₂, EtCO₂: End-tidal CO₂, CO₂: Carbon dioxide, IQR: Interquartile range

Parameter	N	TcCO₂, median (IQR)	EtCO₂, median (IQR)	Z statistic	p-value
Mean CO₂ (mmHg)	127	42.95 (40.50-45.43)	39.00 (34.00-41.00)	-8.06	<0.01
Maximum CO₂ (mmHg)	127	47.40 (45.00-50.83)	44.00 (41.00-46.00)	-7.50	<0.01
Minimum CO₂ (mmHg)	127	36.60 (33.65-38.63)	35.00 (32.00-38.00)	-1.94	0.052

In this study, 10 out of 127 (7.9%) patients were diagnosed with nocturnal hypoventilation based on TcCO₂ monitoring, whereas the EtCO₂ monitoring results of all the above patients did not meet the criteria for nocturnal hypoventilation. In fact, none of the patients in this study (0%) fulfilled the criteria for nocturnal hypoventilation based on EtCO₂ measurements. Therefore, there was a significant discrepancy in the sensitivity of these two modalities.

The agreement between TcCO₂ and EtCO₂ measurements was further assessed using the Bland-Altman analysis across subgroups stratified by demographic characteristics (age, sex) and clinical indicators (OSA severity, obesity, tonsillar hypertrophy, craniofacial abnormalities, and syndromes). The results are summarized in Tables 6-8. The results demonstrated a generally positive bias of TcCO₂ relative to EtCO₂ across all stratified subgroups. Suboptimal agreement was observed in all subgroups for mean, maximum, and minimum TcCO₂ and EtCO₂ measurements, as suggested by the wide limits of agreement. Overall, subgroup analyses suggested relatively stable mean bias and suboptimal agreement across various clinical characteristics. 

**Table 6:** Summary of Bland-Altman analysis results for mean TcCO₂ vs. EtCO₂ of subgroups TcCO₂: Transcutaneous CO₂, EtCO₂: End-tidal CO₂, CO₂: Carbon dioxide, LoA: Limit of agreement, OSA: Obstructive sleep apnea

Category	Subgroup	N	Mean bias	95% CI for mean bias	SD	Lower LoA	Upper LoA	LoA width
Age group (in years)	Three to eight years	34	3.88	1.89, 5.86	5.69	-7.27	15.03	22.30
	Nine to 13 years	56	5.69	3.88, 7.51	6.76	-7.56	18.94	26.50
	14 to 18 years	37	5.89	3.69, 8.08	6.48	-6.81	18.59	25.40
Sex	Female	36	4.25	2.19, 6.31	6.09	-7.69	16.19	23.88
	Male	91	5.67	4.32, 7.02	6.49	-7.05	18.39	25.44
OSA severity	No OSA	38	5.52	2.78, 8.26	8.33	-10.81	21.85	32.66
	Mild	47	4.71	3.10, 6.33	5.49	-6.05	15.47	21.52
	Moderate	16	3.66	1.59, 5.72	3.88	-3.94	11.26	15.20
	Severe	26	6.90	4.56, 9.24	5.78	-4.43	18.23	22.66
Obesity	Present	62	5.19	3.76, 6.62	5.64	-5.86	16.24	22.10
	Absent	65	5.35	3.59, 7.10	7.07	-8.51	19.21	27.72
Tonsillar hypertrophy	Present	68	6.21	4.60, 7.82	6.66	-6.84	19.26	26.10
	Absent	59	4.19	2.64, 5.73	5.93	-7.43	15.81	23.24
Craniofacial abnormality	Present	21	6.71	3.96, 9.46	6.03	-5.11	18.53	23.64
	Absent	106	4.98	3.74, 6.23	6.45	-7.66	17.62	25.28
Syndromic diagnosis	Present	3	7.63	-0.40, 15.67	3.23	1.30	13.96	12.66
	Absent	124	5.21	4.07, 6.36	6.44	-7.41	17.83	25.24

**Table 7:** Summary of Bland-Altman analysis results for maxmium TcCO₂ vs. EtCO₂ of subgroups TcCO₂: Transcutaneous CO₂, EtCO₂: End-tidal CO₂, CO₂: Carbon dioxide, LoA: Limit of agreement, OSA: Obstructive sleep apnea

Category	Subgroup	N	Mean bias	95% CI for mean bias	SD	Lower LoA	Upper LoA	LoA width
Age group (in years)	Three to eight years	34	4.27	2.22, 6.33	5.89	-7.27	15.81	23.08
	Nine to 13 years	56	3.97	2.46, 5.48	5.63	-7.06	15.00	22.06
	14 to 18 years	37	5.14	3.34, 6.95	5.33	-5.31	15.59	20.90
Sex	Female	36	4.01	1.82, 6.21	6.49	-8.71	16.73	25.44
	Male	91	4.53	3.45, 5.61	5.20	-5.66	14.72	20.38
OSA severity	No OSA	38	3.69	1.84, 5.54	5.63	-7.34	14.72	22.06
	Mild	47	3.74	2.24, 5.24	5.11	-6.28	13.76	20.04
	Moderate	16	2.61	0.49, 4.72	3.97	-5.17	10.39	15.56
	Severe	26	7.64	5.17, 10.12	6.13	-4.37	19.65	24.02
Obesity	Present	62	4.50	3.12, 5.89	5.45	-6.18	15.18	21.36
	Absent	65	4.27	2.85, 5.69	5.73	-6.96	15.50	22.46
Tonsillar hypertrophy	Present	68	4.70	3.39, 6.01	5.40	-5.88	15.28	21.16
	Absent	59	4.02	2.51, 5.53	5.78	-7.31	15.35	22.66
Craniofacial abnormality	Present	21	5.91	2.83, 8.99	6.77	-7.36	19.18	26.54
	Absent	106	4.08	3.06, 5.10	5.29	-6.29	14.45	20.74
Syndromic diagnosis	Present	3	6.83	-0.12, 13.79	2.80	1.34	12.32	10.98
	Absent	124	4.32	3.33, 5.32	5.61	-6.67	15.31	21.98

**Table 8:** Summary of Bland-Altman analysis results for minimum TcCO₂ vs. EtCO₂ of subgroups TcCO₂: Transcutaneous CO₂, EtCO₂: End-tidal CO₂, CO₂: Carbon dioxide, LoA: Limit of agreement, OSA: Obstructive sleep apnea

Category	Subgroup	N	Mean bias	95% CI for mean bias	SD	Lower LoA	Upper LoA	LoA width
Age group (in years)	Three to eight years	34	-1.89	-4.56, 0.77	7.64	-16.87	13.09	29.96
	Nine to 13 years	56	1.63	-0.10, 3.36	6.46	-11.03	14.29	25.32
	14 to 18 years	37	3.61	1.08, 6.13	7.46	-11.01	18.23	29.24
Sex	Female	36	0.73	-1.77, 3.24	7.40	-13.77	15.23	29.00
	Male	91	1.49	-0.03, 3.01	7.30	-12.82	15.80	28.62
OSA severity	No OSA	38	1.54	-0.93, 4.02	7.52	-13.20	16.28	29.48
	Mild	47	0.47	-1.95, 2.90	8.26	-15.72	16.66	32.38
	Moderate	16	0.08	-2.91, 3.06	5.61	-10.92	11.08	22.00
	Severe	26	3.07	0.67, 5.47	5.94	-8.57	14.71	23.28
Obesity	Present	62	2.62	0.71, 4.53	7.51	-12.10	17.34	29.44
	Absent	65	-0.01	-1.72, 1.71	6.93	-13.59	13.57	27.16
Tonsillar hypertrophy	Present	68	2.41	0.71, 4.11	7.03	-11.37	16.19	27.56
	Absent	59	-0.03	-1.98, 1.91	7.46	-14.65	14.59	29.24
Craniofacial abnormality	Present	21	1.77	-1.84, 5.39	7.94	-13.79	17.33	31.12
	Absent	106	1.18	-0.21, 2.57	7.21	-12.95	15.31	28.26
Syndromic diagnosis	Present	3	6.07	-4.52, 16.65	4.26	-2.28	14.42	16.70
	Absent	124	1.16	-0.15, 2.46	7.34	-13.23	15.55	28.78

The correlation between TcCO₂ and EtCO₂ showed some degree of variability across the different clinical and demographic subgroups (Table 9). When considering age, slightly higher correlations were noted in the three-to-nine-year age group for maximum values (rs = 0.413, p = 0.015) and in the nine-to-14-year age group for minimum values (rs = 0.275, p = 0.041). In the sex-based analysis, significant correlations were primarily observed in male participants, particularly for maximum CO₂ levels (rs = 0.397, p < 0.001). Regarding sleep-disordered breathing, weak correlations for maximum CO₂ were found in patients with no OSA (rs = 0.393, p = 0.015) and mild OSA (rs = 0.393, p = 0.006), though these trends were not maintained in moderate or severe cases. The presence of tonsillar hypertrophy appeared to be associated with marginally stronger correlations for mean (rs = 0.242, p = 0.047) and maximum (rs = 0.261, p = 0.032) values compared to those without the condition. Conversely, the relationship seemed less apparent in patients with craniofacial abnormalities or syndromic diagnoses. Obesity did not appear to be a major factor, as significant results for maximum values were limited to the non-obese subgroup (rs = 0.318, p = 0.010). Overall, the subgroup analysis suggested only inconsistent and weak correlations between the two modalities, indicating that these results should be interpreted with caution.

**Table 9:** Spearman correlation between TcCO2 and EtCO2 measurements of subgroups rs: Spearman's rank correlation coefficient, TcCO₂: Transcutaneous CO₂, EtCO₂: End-tidal CO₂, CO₂: Carbon dioxide, OSA: Obstructive sleep apnea *indicates p < 0.05; * indicates p < 0.01

Category	Subgroup	N	Correlation TcCO^2^vs. EtCO_2_	rs	p-value
Age group (in years)	Three to nine	34	Mean	0.262	0.135
			Maximum	0.413	0.015*
			Minimum	0.132	0.458
	Nine to 14	56	Mean	0.194	0.152
			Maximum	0.143	0.294
			Minimum	0.275	0.041*
	15 to 18	37	Mean	0.122	0.474
			Maximum	0.370*	0.024*
			Minimum	0.075	0.659
Sex	Female	36	Mean	0.047	0.784
			Maximum	0.081	0.637
			Minimum	0.003	0.987
	Male	91	Mean	0.244	0.020*
			Maximum	0.397	< 0.001**
			Minimum	0.143	0.177
OSA severity	None	38	Mean	0.207	0.212
			Maximum	0.393	0.015*
			Minimum	0.007	0.968
	Mild	47	Mean	0.264	0.073
			Maximum	0.393	0.006**
			Minimum	0.117	0.434
	Moderate	16	Mean	-0.028	0.917
			Maximum	0.026	0.924
			Minimum	0.033	0.905
	Severe	26	Mean	0.049	0.811
			Maximum	0.146	0.476
			Minimum	0.270	0.182
Obesity	Present	62	Mean	0.176	0.172
			Maximum	0.236	0.064
			Minimum	0.123	0.342
	Absent	65	Mean	0.147	0.244
			Maximum	0.318	0.010**
			Minimum	0.140	0.268
Tonsillar hypertrophy	Present	68	Mean	0.242	0.047*
			Maximum	0.261	0.032*
			Minimum	0.167	0.173
	Absent	59	Mean	0.108	0.414
			Maximum	0.296	0.023*
			Minimum	0.009	0.948
Craniofacial abnormality	Present	21	Mean	-0.102	0.659
			Maximum	-0.023	0.922
			Minimum	0.137	0.553
	Absent	106	Mean	0.232	0.017*
			Maximum	0.352	< 0.001**
			Minimum	0.109	0.266
Syndromic diagnosis	Present	3	Mean	0.500	0.667
			Maximum	0.866	0.333
			Minimum	0.500	0.667
	Absent	124	Mean	0.163	0.070
			Maximum	0.288	0.001**
			Minimum	0.107	0.238

Statistical evaluation using the Wilcoxon signed-rank test identified a systematic and significant difference between TcCO₂ and EtCO₂ across the majority of patient subgroups (Table 10). Across nearly all categories, EtCO₂ measurements demonstrated significantly lower median values compared to TcCO₂ (p < .001). This downward deviation in EtCO₂ relative to TcCO₂ was most pronounced in the mean CO₂ and maximum CO₂ parameters.

**Table 10:** Wilcoxon signed-rank test comparison of TcCO2 vs. EtCO2 measurement of subgroups *indicates p < 0.05; **indicates p < 0.01 TcCO₂: Transcutaneous CO₂, EtCO₂: End-tidal CO₂, CO₂: Carbon dioxide, OSA: Obstructive sleep apnea, IQR: Interquartile range

Category	Subgroup	N	Parameter	TcCO₂median (IQR)	EtCO₂ median (IQR)	Z-score	p-value
Age group (in years)	Three to eight	34	Mean CO₂	41.950 (39.43-46.10)	38.50 (36.00-42.25)	-3.449	< 0.001**
			Maximum CO₂	47.150 (44.75-51.93)	44.00 (41.00-47.25)	-3.907	< 0.001**
			Minimum CO₂	34.000 (30.85-38.63)	36.50 (33.00-38.55)	-1.641	0.101
	Nine to 13	56	Mean CO₂	43.000 (40.83-45.38)	39.00 (34.00-41.00)	-5.649	< 0.001**
			Maximum CO₂	47.250 (45.13-51.55)	44.00 (41.25-47.75)	-4.424	< 0.001**
			Minimum CO₂	36.550 (34.33-37.85)	34.50 (32.00-38.00)	-1.793	0.073
	14 to 18	37	Mean CO₂	44.400 (40.25-45.55)	39.00 (33.00-41.00)	-4.504	< 0.001**
			Maximum CO₂	47.400 (45.00-50.40)	43.00 (39.50-45.50)	-4.579	< 0.001**
			Minimum CO₂	38.600 (34.05-39.60)	34.00 (30.50-38.00)	-3.138	0.002**
Sex	Female	36	Mean CO₂	41.650 (39.78-45.23)	39.50 (35.00-42.00)	-3.260	0.001**
			Maximum CO₂	46.750 (43.78-49.15)	43.00 (41.00-46.00)	-2.970	0.003**
			Minimum CO₂	35.700 (33.55-38.65)	35.00 (32.25-38.00)	-0.699	0.484
	Male	91	Mean CO₂	43.600 (40.60-45.60)	38.00 (34.00-41.00)	-7.245	< 0.001**
			Maximum CO₂	47.500 (45.40-51.40)	44.00 (41.00-47.00)	-6.887	< 0.001**
			Minimum CO₂	36.700 (33.40-39.00)	34.00 (31.00-38.00)	-1.835	0.067
OSA severity	No OSA	38	Mean CO₂	42.400 (39.88-45.08)	39.00 (33.75-42.00)	-3.814	< 0.001**
			Maximum CO₂	45.450 (43.63-48.58)	43.00 (41.00-46.00)	-3.327	< 0.001**
			Minimum CO₂	35.300 (32.55-38.25)	35.00 (30.00-38.00)	-1.313	0.189
	Mild	47	Mean CO₂	42.600 (40.30-45.90)	38.00 (35.00-41.00)	-5.042	< 0.001**
			Maximum CO₂	46.700 (44.80-50.30)	44.00 (41.00-47.00)	-4.487	< 0.001**
			Minimum CO₂	36.700 (33.40-39.10)	36.00 (32.00-38.00)	-0.191	0.848
	Moderate	16	Mean CO₂	43.700 (41.30-45.15)	40.00 (37.25-42.00)	-2.819	0.005**
			Maximum CO₂	47.900 (46.45-50.73)	45.50 (43.00-48.00)	-2.120	0.034
			Minimum CO₂	37.050 (33.65-38.35)	34.00 (32.25-38.00)	-0.233	0.816
	Severe	26	Mean CO₂	44.500 (40.98-46.10)	38.00 (33.00-41.00)	-4.115	< 0.001**
			Maximum CO₂	50.950 (46.90-53.98)	43.50 (40.00-46.75)	-4.178	< 0.001**
			Minimum CO₂	37.300 (34.28-38.93)	33.50 (29.50-36.25)	-2.248	0.025*
Obesity	Present	62	Mean CO₂	42.600 (39.98-45.30)	38.00 (33.75-41.00)	-5.782	< 0.001**
			Maximum CO₂	47.100 (45.08-50.35)	43.00 (41.00-46.00)	-5.420	< 0.001**
			Minimum CO₂	36.650 (34.15-38.93)	34.00 (31.00-38.00)	-2.826	0.005**
	Absent	65	Mean CO₂	43.200 (40.55-45.70)	39.00 (35.00-42.00)	-5.584	< 0.001**
			Maximum CO₂	47.600 (44.80-51.90)	44.00 (41.00-48.50)	-5.130	< 0.001**
			Minimum CO₂	36.500 (31.95-38.65)	36.00 (32.00-38.00)	-0.037	0.971
Craniofacial abnormality	Present	21	Mean CO₂	45.200 (41.10-45.85)	38.00 (33.00-41.00)	-3.883	< 0.001**
			Maximum CO₂	49.300 (45.90-51.20)	42.00 (39.00-49.50)	-3.302	0.001**
			Minimum CO₂	37.700 (34.50-38.80)	35.00 (31.50-37.50)	-1.078	0.281
	Absent	106	Mean CO₂	42.800 (40.38-45.33)	39.00 (35.00-41.00)	-7.163	< 0.001**
			Maximum CO₂	47.050 (44.80-50.65)	44.00 (41.00-46.00)	-6.749	< 0.001**
			Minimum CO₂	36.450 (33.33-38.83)	35.00 (31.75-38.00)	-1.637	0.102
Syndromic diagnosis	Present	3	Mean CO₂	41.500 (36.90-44.50)	33.33 (32.00-35.00)	-1.633	0.102
			Maximum CO₂	45.600 (45.40-50.50)	40.33 (37.00-42.00)	-1.604	0.109
			Minimum CO₂	37.500 (30.00-37.70)	29.00 (27.00-32.00)	-1.604	0.109
	Absent	124	Mean CO₂	43.000 (40.50-45.58)	39.00 (35.00-41.00)	-7.893	< 0.001**
			Maximum CO₂	47.400 (44.85-50.98)	44.00 (41.00-46.75)	-7.314	< 0.001**
			Minimum CO₂	36.550 (33.55-38.88)	35.00 (32.00-38.00)	-1.701	0.089

In the age group analysis, EtCO₂ was significantly lower than TcCO₂ for mean and maximum values among all age groups. Notably, while the two modalities showed no significant difference in minimum CO₂ for the age groups three to eight years and nine to 13 years, a significant discrepancy emerged in the 14 to 18 years age group (Z = -3.14, p = .0002). This measurement bias persisted regardless of sex, obesity status, tonsillar hypertrophy, or craniofacial abnormalities (all p < 0.001). Regarding OSA severity, the difference in mean CO₂ remained consistent from the no OSA group (Z = -3.81) through the severe group (Z = -4.12). In patients with severe OSA, the discrepancy extended to the minimum CO₂ values (p = 0.025), suggesting that the gap between the two modalities increases with disease severity.

Patient safety

No adverse events related to TcCO₂ monitoring were reported, including skin injury or thermal burns from prolonged sensor application. These findings demonstrate the clinical safety of TcCO₂ monitoring in the pediatric population.

## Discussion

Our primary objective revealed that TcCO₂ measurements were systematically higher than EtCO₂ measurements. For comparison of mean CO₂ values, the mean bias exceeded the predefined criteria of clinical interchangeability of within ±5 mmHg, whereas for comparison of maximum and minimum CO₂ values, the mean bias was within the predefined criteria of clinical interchangeability of within ±5 mmHg. Critically, the limits of agreement exceeded the clinically acceptable threshold of ±5 mmHg across all parameters, including mean, maximum, and minimum values. The relatively broad limits of agreement suggested that the level of agreement between the two modalities may not be sufficient for one to reliably replace the other in a clinical setting. Regarding the secondary objectives, a weak but statistically significant positive correlation was observed between maximum TcCO₂ and EtCO₂. Despite this weak correlation, TcCO₂ values remained consistently higher, with statistically significant differences noted for both mean and maximum CO₂ values. Further statistical analysis of various subgroups stratified by demographic characteristics (age and sex) and clinical indicators (OSA severity, obesity, tonsillar hypertrophy, craniofacial abnormalities, and syndromes) yielded similar findings. Additionally, TcCO₂ and EtCO₂ provided different perspectives on the diagnosis of nocturnal hypoventilation. While 10 patients met the criteria of nocturnal hypoventilation via TcCO₂ monitoring, these cases were not captured by EtCO₂ monitoring. It is also worth mentioning that EtCO₂ measurements were unsuccessful for a small subset of the study population (11 out of 138), in contrast to TcCO₂ measurements, which did not encounter similar technical issues. Overall, our findings suggested that the two methods may not be fully interchangeable in pediatric PSG, and clinicians should be mindful of these variations when interpreting the results.

The difference observed between TcCO₂ and EtCO₂ measurements in this study could be explained by the distinct physiological mechanisms underlying the two monitoring methods. The EtCO₂ monitoring measures the PaCO₂ in exhaled gas at the end of expiration and is intended to approximate alveolar CO₂ tension, and thus PaCO₂, in ideal conditions. However, in reality, EtCO₂ values are influenced by several physiological factors, including ventilation-perfusion (V/Q) relationships and the presence of anatomical and physiological dead space. The presence of physiological dead space and V/Q mismatch can lead to dilution of alveolar gas during expiration, resulting in EtCO₂ values that are lower than arterial CO₂ levels [7]. In contrast, TcCO₂ monitoring estimates CO₂ tension by measuring the diffusion of CO₂ across the skin using a heated electrode. The heating of the sensor increases local perfusion and facilitates diffusion of carbon dioxide from arterialized capillary blood, allowing TcCO₂ measurements to approximate PaCO₂ more closely [7]. As a result, TcCO₂ values are often slightly higher than EtCO₂ measurements.

Our findings were generally consistent with previous studies comparing TcCO₂ and EtCO₂ monitoring. A 2006 study by Kirk et al. reported the maximum and mean CO₂ measurements obtained by TcCO₂ and EtCO₂ in close agreement [18]. These findings diverge from our observations, a discrepancy that may be attributed to variations in patient demographics and study protocols. A more recent study by Jurado et al., however, demonstrated that in children aged less than three years old, TcCO₂ was superior to EtCO₂ in the detection of nocturnal hypoventilation [9]. Although the latter study focused on a younger demographic compared to our study population, their conclusions are consistent with our findings. Furthermore, in another recent study by Park et al., including 55 adults, TcCO₂ monitoring values (mean, minimum, and maximum) were higher than EtCO₂ monitoring [10]. Previous studies involving pediatric patients on invasive ventilation across various clinical settings, including intensive care and postoperative monitoring, had explored these modalities [19-22]. In these settings, TcCO₂ and EtCO₂ were typically compared against PaCO₂ values obtained via arterial blood gas (ABG) analysis. Several of these reports suggested a closer degree of concordance between TcCO₂ and PaCO₂ than that observed with EtCO₂, although considerable variability in the data was often noted. These observations may suggest that TcCO₂ aligns more closely with PaCO₂ in certain contexts, though the wide dispersion of values indicated that neither method served as a perfect substitute for PaCO₂ measurements. In conclusion, our findings are consistent with the current literature and offer insights into the diagnostic discordance between these two CO₂ monitoring modalities within a pediatric cohort that reflects general population characteristics.

Based on our findings, TcCO₂ measurement may appear more sensitive than EtCO₂ for detecting nocturnal hypoventilation. However, without ABG as a reference standard, it was not possible to determine the true diagnostic accuracy of either modality, and this observation warranted further investigation. Nevertheless, EtCO₂ may offer complementary value when utilized simultaneously with TcCO₂ monitoring. It could serve as a secondary reference to identify potentially inaccurate TcCO₂ readings resulting from sensor drift or other technical artifacts. In case additional EtCO₂ monitoring is performed in sleep laboratories, it is preferable to have clinical staff readily available to attend to the patients and promptly address related technical issues, such as dislodgement of the nasal cannula.

Limitations

This study had several limitations. First, as a single-center study, the findings may have limited generalizability. Second, TcCO₂ monitoring was subjected to sensor drift, which may result in overestimation of CO₂ levels; although drift-corrected values were used, residual measurement error could not be excluded. In addition, sleep stage-specific CO₂ analysis was not feasible, as TcCO₂ measurements could not be precisely synchronized with 30-second PSG epochs due to physiological lag. As a result, differentiation between rapid eye movement (REM)- and non-rapid eye movement (NREM)-related hypoventilation was not possible.

Another limitation is related to the observed discrepancy in hypoventilation detection between modalities, with cases identified by TcCO₂ but not by EtCO₂. This may reflect a combination of TcCO₂ overestimation and EtCO₂ underdetection. End-tidal CO₂, as a measure of end-expiratory gas, is susceptible to ventilation-perfusion mismatch, dead space, and sampling limitations (e.g., mouth breathing or leak), which may lead to underestimation of PaCO₂ [7]. Conversely, TcCO₂ may be influenced by factors such as sensor drift and skin perfusion. Therefore, this discrepancy should be interpreted cautiously, as it may reflect both methodological limitations and physiological differences between measurement techniques. The exclusion of cases with failed EtCO₂ recordings may have introduced selection bias by restricting the analysis to technically successful measurements, thereby limiting the generalizability of the findings and constraining interpretation of the comparison between modalities.

Finally, the absence of ABG measurements as a reference standard limited the ability to determine the absolute accuracy of TcCO₂ and EtCO₂. As a result, this study could not establish which modality more closely reflected true arterial PaCO₂ levels, and comparisons were therefore limited to relative differences between methods. While ABG remains the gold standard for assessing PaCO₂, its use in pediatric PSG is limited by its invasive nature, the provision of only intermittent measurements, and the potential to disrupt sleep and alter physiological parameters [7]. Consequently, ABG sampling was not routinely performed in our setting. The findings, therefore, reflect real-world clinical practice, in which non-invasive CO₂ monitoring modalities are preferentially used.

## Conclusions

While both EtCO₂ and TcCO₂ are utilized in pediatric PSG, our findings indicated they were not interchangeable. The suboptimal agreement, higher average TcCO₂ readings, and lack of significant correlation suggested distinct performance profiles for each modality. Furthermore, without ABG data as a reference standard, the comparative accuracy of these methods in reflecting true arterial CO₂ levels or their reliability in diagnosing nocturnal hypoventilation remained undetermined. Future studies incorporating blood gas analysis may help provide a more comprehensive comparison against a reference standard. Continued advancements in monitoring techniques may further improve the assessment and management of sleep-disordered breathing in children.
